# Challenging reconstruction of oncologic defects of the parotid region: a single-institution experience and decision-making framework proposal

**DOI:** 10.1007/s10006-026-01541-9

**Published:** 2026-03-30

**Authors:** Andrea Cassoni, Resi Pucci, Nicolò Mangini, Angelica Meriano, Angela Palma, Ashleigh Weyh, Rui P. Fernandes, Marco Della Monaca, Valentino Valentini

**Affiliations:** 1 Department of Oral and Maxillofacial Sciences, Sapienza University of Rome, Rome, Italy; 2 Maxillofacial Surgery Unit, San Camillo-Forlanini Hospital, Rome, Italy; 3 Department of Oral and Maxillofacial Surgery, University of Illinois Chicago, Chicago, IL USA; 4 Department of Oral and Maxillofacial Surgery, University of Florida Health, Jacksonville, FL USA

**Keywords:** Parotid cancer, Reconstruction, Local flaps, Locoregional flaps, Free flaps, Nerve reconstruction

## Abstract

**Objectives:**

Reconstruction of the parotid region presents challenges due to the area’s complex anatomy. A classification of defects from ablative surgery can simplify and systematize the reconstructive approach. This study aims to propose a structured decision-making framework to guide reconstructive planning.

**Materials and methods:**

A retrospective study was conducted on patients with malignant parotid tumors treated at Policlinico Umberto I between 2013 and 2023. Non-malignant tumors were excluded. The analysis focused on the involvement of skin, soft tissue, bone, and nerves, the type of parotidectomy (superficial, total, extended), whether neck dissection was performed, and the type of reconstruction used.

**Results:**

Sixty-seven patients (mean age 55 ± 16 years) were included. Primary closure was performed in 42 cases (62.7%). Thirteen patients (19.4%) required reconstruction of soft tissue and/or bony defects, including local cervicofacial flaps (*n* = 2), pedicled locoregional flaps (*n* = 4), free flaps (*n* = 2), deep circumflex iliac artery free flaps (*n* = 2), and pectoralis major myocutaneous flaps (*n* = 3), with one secondary free scapular flap after iliac crest failure. Facial nerve resection was necessary in 25 patients (37%). Immediate facial nerve reconstruction was achieved with direct neurorrhaphy or interposition grafting in 11 cases and with nerve transfers (masseteric-to-facial or hypoglossal-to-facial) in 5 cases. Static facial reanimation procedures were performed in 9 patients, either alone or in combination.

**Conclusions:**

Successful parotid reconstruction focuses on minimizing skin tension, addressing color mismatch, restoring volume, and preserving nerve function. Based on this experience, we propose a practical decision-making framework tailored to defect volume and functional loss involving skin/soft tissue, bone, and nerve.

## Introduction

Major salivary gland malignant tumors (MaSGMT) are uncommon, despite being of great interest to surgeons. The annual incidence of these tumors is 6–11 in the parotid per 10^6 population [1, 2].

The reconstruction of the parotid region represents a challenge for the reconstructive surgeon since defects can be complex and the aesthetic and functional result will have a great impact on patients’ lives. Given the complex anatomy of the region, surgical defects from parotid malignancy vary greatly based on tumor extension. Resection may involve the skin, soft tissues, underlying structures, bone (mandible, maxilla, zygomatic arch), and the facial nerve; in cases of large tumors, it can also affect the ear canal and skull base.

The difficulties encountered when skin resection is required are wound closure and skin tension if the resection is wide, and a high likelihood of scarring. When the resection includes deep tissues, such as subcutaneous tissue and muscles, it can lead to a significant depression of the surgical site and therefore alteration in facial contour and symmetry [1]. Resection of just a small portion of bone may be inconsequential and may not require reconstruction, whereas wider resections can lead to alterations in facial contouring, in oral occlusion, as well as mastication and speech. A facial nerve lesion, depending on the branches involved, can cause extensive paralysis of facial mimic muscles [2].

While the fundamental aim of ablative surgery is to achieve negative oncological margins [3–7], the objectives of reconstructive surgery are morpho-functional restoration, rapid recovery and reduction of complications, especially since a majority of patients will have to undergo adjuvant radiotherapy as part of their cancer treatment [7]. Rapid healing of the surgical site is important to avoid delays in starting post-operative radiotherapy treatments [8, 9]. Functional and aesthetic restoration is a key component of reconstructive surgery, especially the craniomaxillofacial region. A better aesthetic result offers a better quality of life for the patient, a reduced impact on their personal, social and working life, and an overall reduced psychological impact postoperatively [10].

Special attention is given to facial nerve management. According to international guidelines and recommendations, every effort is made to preserve the facial nerve when not infiltrated [1–4]. When there is gross involvement, the facial nerve is resected for oncologic safety [6]; and the remaining stumps, when cancer-free, can be used to reconstruct the continuity of the nerve [6, 11].

Considering the difficulties of such reconstruction [1], the lack of standardized guidelines and the existence of multiple published algorithms available for reference in international literature, this makes reconstructive decision making a difficult problem for surgeons.

This study takes into consideration the histological type of the tumor, the type of ablative surgery performed [12], and the type of defect obtained. The study aims to provide a structured decision-making framework for reconstruction which consists in the schematic analysis of the deficit to identify the main issues and to address them correctly with a surgical plan. The reconstructive framework used considers the tridimensionality of the defect (missing bone, soft tissue, nerve) and is based upon two main criteria: type of resected tissue and volume of the defect.

This study analyzes a single-institution cohort of patients with malignant parotid tumors, focusing on the type of resected tissues and reconstructive strategies. Based on these data, we propose an algorithm considering skin/soft tissue, bone, and nerve involvement, as well as defect volume.

## Materials and methods

A single-institution, retrospective observational study was conducted. The inclusion criteria were: (1) patients with malignant primary tumors of the parotid gland, (2) surgically treated in the Department of Oral and Maxillofacial Sciences, Sapienza University of Rome, Italy, (3) between January 2013 and December 2023, and (4) with complete clinical and radiographic documentation. All patients gave their informed consent for inclusion. The study was approved by the Ethics Committee of Sapienza University of Rome, Italy (Prot. n. 0000209).

All patients with malignant tumors of the parotid gland, including tumors with different origins and histology, were reviewed. For each patient the following data were gathered: lesion site, histology, staging, surgical procedure, comorbidities, healing time and complications. Each case was analyzed for the presence of cutaneous, soft tissues, bony and neural invasion; type of parotidectomy (superficial, total, radical, radical extended to surrounding tissues), neck dissection, and type of reconstruction performed. Variables describing the patient demographics were recorded.

Indications for neck dissection were defined according to NCCN guidelines [13] for salivary gland malignancies and TNM staging according to the 8th edition of the American Joint Committee on Cancer (AJCC) [14], based on preoperative clinical and radiological assessment, tumor grade, T stage, and multidisciplinary tumor board discussion. Neck dissection was routinely performed in clinically or radiologically node-positive (cN+) patients and in high-grade and/or T3–T4 tumors, in accordance with NCCN recommendations.

In this retrospective analysis, reconstructive success was defined by the achievement of wound closure without complications, recovery of function (e.g., facial symmetry or oral competence), and sufficient healing time to allow timely adjuvant therapy.

Patients were divided into groups according to the type of defect, determined by the ablative surgery: (1) skin/soft tissue defects; (2) bone defects; (3) facial nerve invasion. The primary outcome assessed was the type of reconstruction performed, while secondary descriptive outcomes included wound healing, need for revision surgery, and functional recovery as documented in clinical follow-up.

**Fig. 1:**
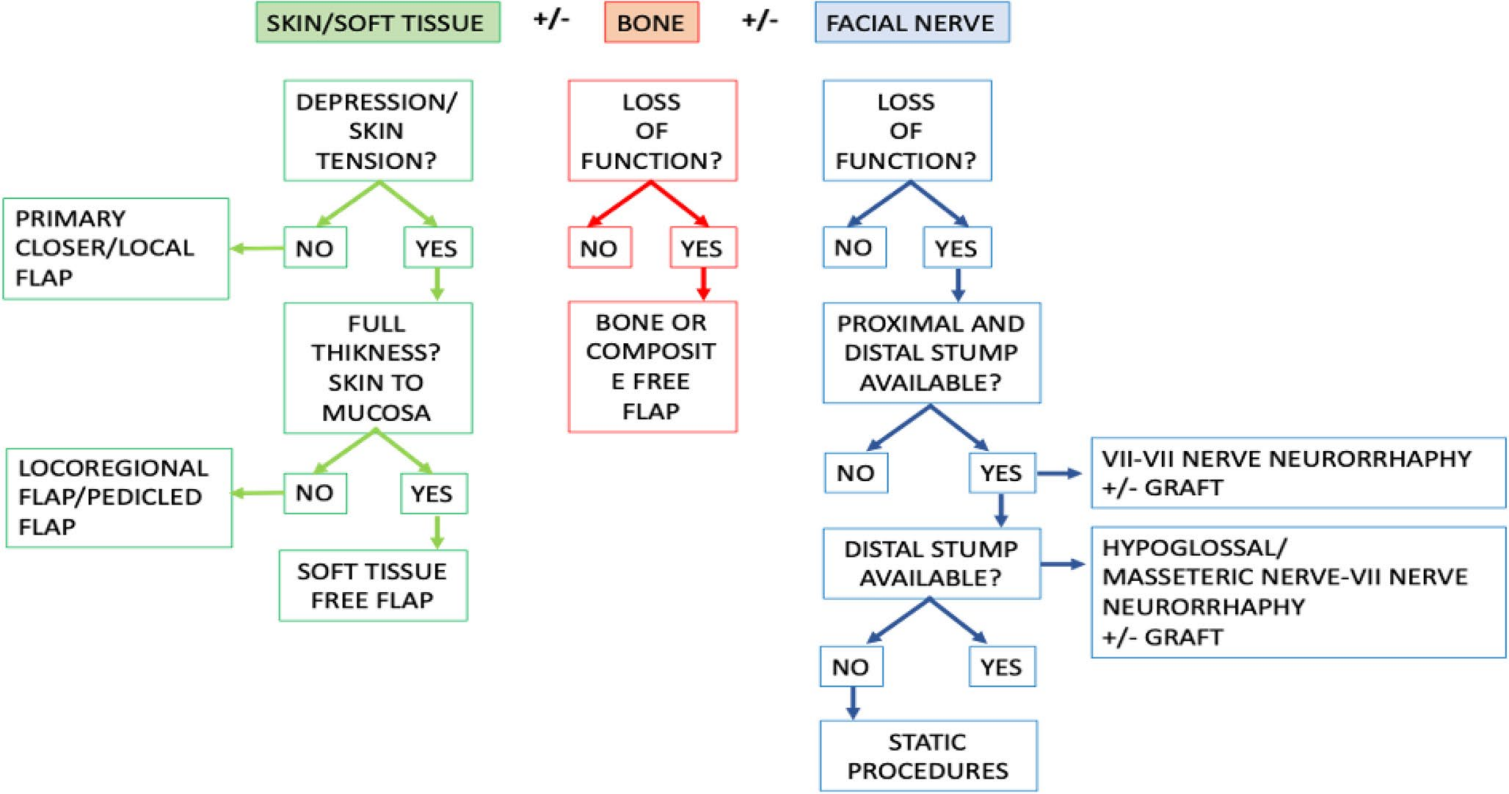
Reconstructive algorithm for parotid defects: schematic flowchart illustrating decision-making based on (1) resected tissue: skin/soft tissue, bone, facial nerve; and (2) defect volume and depth. Paths lead to options ranging from primary closure and local flaps to pedicled or free flaps, and nerve reconstruction or static reanimation

**Fig. 2:**
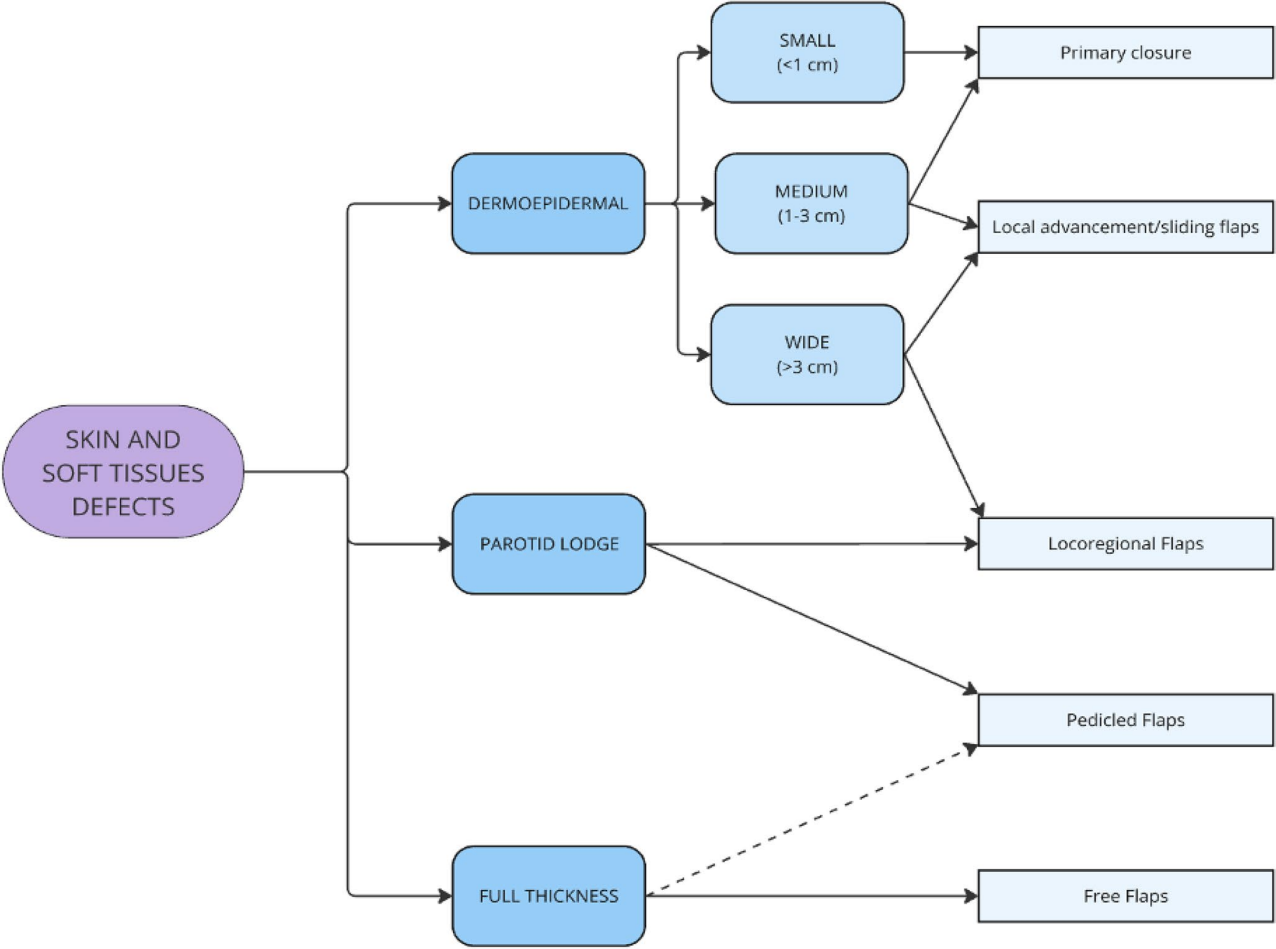
Skin/soft tissue defect classification and reconstructive options: tiered breakdown between (**a**) superficial defects (small <1 cm, medium 1–3 cm, wide >3 cm) guiding toward primary closure, local flaps, or locoregional/free flaps; (**b**) volumetric soft tissue deficits requiring volume tissue transfer

The reconstructive algorithm (Fig. 1) is based on (1) the type of resected tissues (skin/soft tissues, bone, nerve) and (2) the volume dimensions of the defect. The reconstructive options resulting from the application of the presented algorithm must then be interpreted considering the characteristics of the patient, the probability of success, and the need for postoperative radiotherapy. As for skin/soft tissues defects, we can make a distinction between (1) superficial defects and (2) volumetric defects. Superficial defects are further classified into (1) small (< 1 cm), (2) medium (1–3 cm) and (3) wide (> 3 cm) defects using Dobratz and Hilger classification of cheek skin defects [11]. For volumetric defects, a distinction can be made between (1) a resection that includes only the gland, (2) one that involves subparotid muscles (parotid lodge) and (3) a defect extended to the oral mucosa (Fig. 2).

**Fig. 3:**
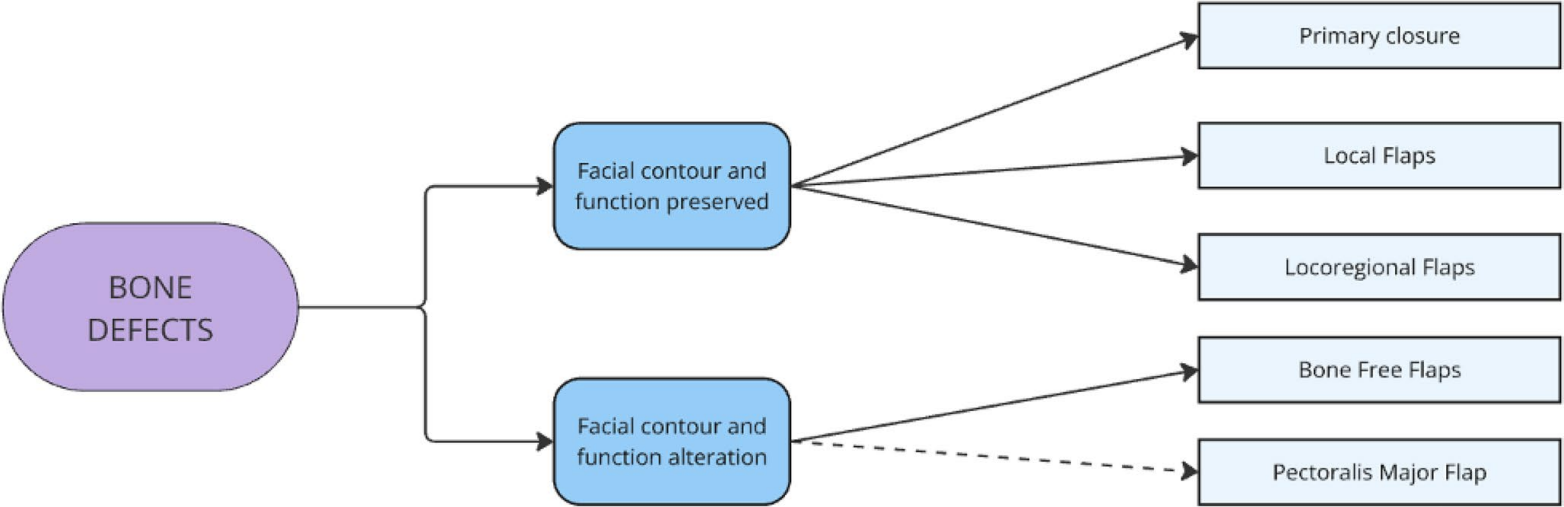
Bone defect stratification and reconstruction: differentiates between—(**a**) bone resections not impairing function/aesthetics (no reconstruction needed) and (**b**) composite defects requiring reconstruction using free bone flaps (e.g., iliac crest) or pedicled muscles (e.g., pectoralis major) when microsurgery is contraindicated

**Fig. 4:**
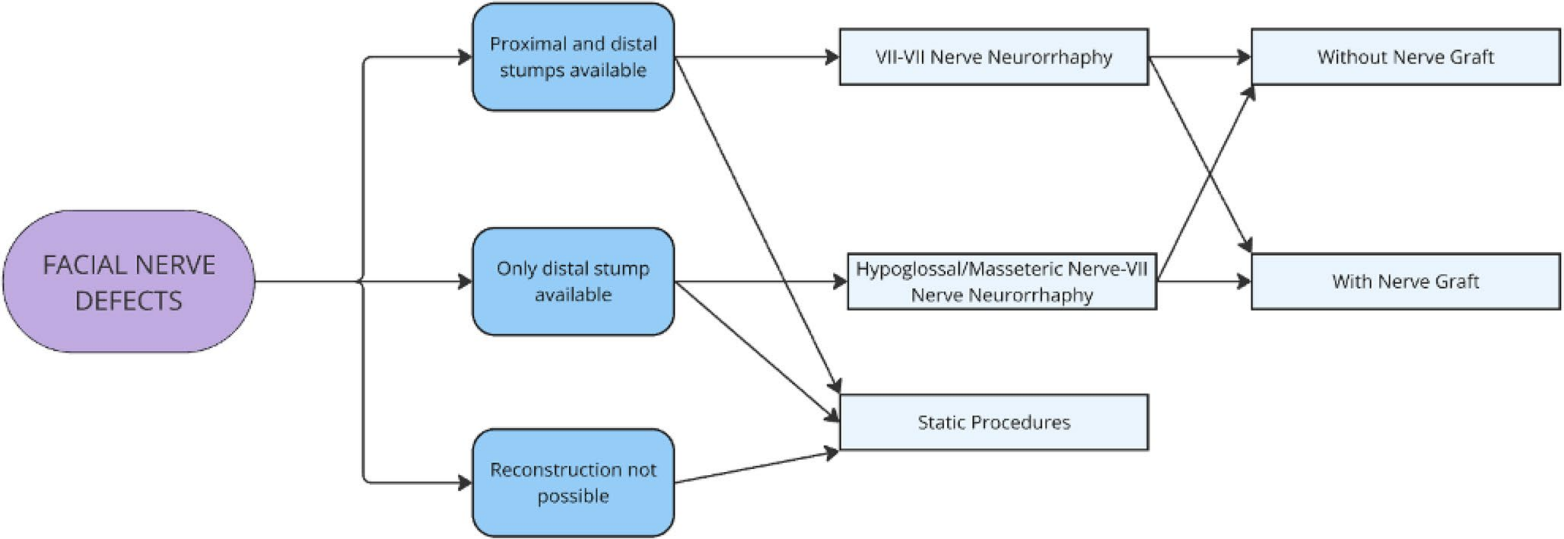
Facial nerve defect algorithm: delineates strategy based on nerve stump availability—reconstruct continuity via neurorrhaphy or graft if trunk available; use masseteric or hypoglossal nerve for reinnervation if only distal branches are present; apply static procedures when nerve reconstruction is not feasible

Bone defects are further subdivided according to whether (1) they cause an impairment in facial aesthetics and function or (2) they do not (Fig. 3). Finally, facial nerve defects were differentiated according to whether (1) the proximal trunk and distal branches were preserved or (2) they were not (Fig. 4).

Based on this classification, the most appropriate reconstructive technique was selected for each case, following the proposed algorithms and considering patient-specific factors such as age, comorbidities, and the need for adjuvant therapy.

## Results

A total of 67 patients (35 female, 32 male; mean age 55 ± 16 years) were included. The most common histologic types were salivary duct carcinoma (15.5%) and myoepithelial carcinoma (low grade: 15.5%; high grade: 9%; intermediate grade: 7%), followed by squamous cell carcinoma (12%), acinic cell carcinoma (10%), adenoid-cystic carcinoma (10%), myoepithelial-epithelial carcinoma (10%). Less common histologic types were myoepithelial carcinoma (5%), mammary analogue secretory carcinoma (one case), pleomorphic adenocarcinoma (two cases), carcinoma ex pleomorphic adenoma (one case), Hodgkin lymphoma (one case), and malignant fibrous solitary tumor (one case).

Reconstructive techniques were selected according to the defect classification proposed in our algorithmic framework, and adapted based on individual clinical features.

As shown in Table 1: 34 patients (50.7%) underwent total parotidectomy, 22 (32.8%) superficial parotidectomy and 11 (16.4%) patients underwent total parotidectomy with sacrifice of the VII cranial nerve. In 40% of the patients (*n* = 27) a radical or radical modified ipsilateral neck dissection (levels I–V) was performed; in 23% (*n* = 17) an elective ipsilateral neck dissection was performed, of which six patients had elective neck dissection (END) levels I–IV, four patients END levels Ib–V, two patients Ib–Va, one patient II–V, one patient IIb–V, one IIb, one II–IV, one Ib IIa III Va.

**Table 1:** Details of ablative surgery

Surgical procedure	*n* (%)
Type of parotidectomy	
• Total parotidectomy	34 (50.7)
• Superficial parotidectomy	22 (32.8)
• Total parotidectomy with VII nerve sacrifice	11 (16.4)
**Neck dissection**	
• Radical or radical modified ipsilateral (levels I–V)	27 (40.0)
• Elective ipsilateral	17 (25.0)
– Levels I–IV	6
– Levels Ib–V	4
– Levels Ib–Va	2
– Levels II–V	1
– Level IIb–V	1
– Level IIb	1
– Levels II–IV	1
– Levels Ib, IIa, III, Va	1
• No neck dissection	23 (34.3)

Percentages are calculated on a total of 67 patients

In 34.3% of cases (*n* = 23), neck dissection was not performed, as these patients did not meet NCCN criteria for elective neck treatment.

As shown in Table 2, reconstructive techniques included: in most patients (42 cases, 62.7%), primary closure was performed achieving a good aesthetic and functional outcome. In 25 cases (37.3%), a reconstructive technique was necessary. For soft tissue-only defects, local cervicofacial and retroauricular sliding/rotation flaps were used in two patients, a laterocervical skin graft was used in one patient, and a supraclavicular flap was used in one patient [15]. A pedicled pectoralis major flap was used in four patients, a free myocutaneous latissimus dorsi flap in one patient, and a free anterolateral thigh (ALT) flap in one patient. For composite soft tissue and bony defects, reconstruction with osteomuscular or osteomyocutaneous chimeric free flap of iliac crest was performed in two patients, and in one patient the reconstruction with iliac crest was not successful and a second surgery was performed with a free scapular flap. Facial nerve reconstruction was performed in addition to soft tissue or bony reconstruction when indicated, and therefore some patients underwent combined reconstructive procedures.

**Table 2:** Reconstruction techniques

Reconstruction method	*n*
**Primary closure**	42
**Reconstructive procedures**	13
• Local cervicofacial/retroauricular flaps	2
• Laterocervical skin graft	1
• Supraclavicular flap	1
• Pedicled pectoralis major flap	4
• Free myocutaneous latissimus dorsi flap	1
• ALT flap	1
• Iliac crest osteomuscular/osteomyocutaneous flap	2
• Free scapular flap (after failed iliac crest reconstruction)	1
**Facial nerve reconstruction**	25
• Facial nerve reconstruction (VII-VII neurorraphy)	11
• Facial nerve reconstruction (hypoglossal/masseteric-VII neurorraphy)	5
• Static reanimation (alone or combined)	9

*ALT * = anterolateral thigh, values indicate number of patients

Facial nerve resection was required in 25 patients (37%) due to macroscopic tumor involvement. In 11 cases, this corresponded to sacrifice of the main trunk of the facial nerve during total parotidectomy, whereas in the remaining 14 patients tumor infiltration necessitated resection of one or more peripheral branches.

When both proximal and distal nerve stumps were available, immediate nerve reconstruction was performed during the same surgical procedure. In these cases, nerve continuity was restored by direct termino-terminal neurorrhaphy or by interposition nerve grafting, according to the length of the defect.

In 5 patients in whom direct reconstruction was not feasible, nerve transfer procedures were undertaken based on intraoperative findings. Specifically, three patients underwent masseter-to-facial nerve transfer, while two patients were treated with hypoglossal-to-facial nerve transfer.

In 9 patients, dynamic reconstruction was not indicated and static facial reanimation procedures were performed to improve facial symmetry and ocular protection. These included upper eyelid loading for lagophthalmos management, fascial suspension techniques for commissural support, and soft-tissue augmentation procedures such as lipofilling, tailored to individual reconstructive requirements.

## Discussion

This paper describes the experience of more than twenty years of our institution in treating parotid malignancies and in the reconstruction of the resulting defects. This approach combines evaluation of the ablative defect with patient-specific variables. A systematic analysis allows classification and more precise surgical planning. The main characteristics we consider are the type of resected tissue (skin and/or soft tissues, bone, facial nerve) and dimensions of the defect (superficial extension and extension in depth) (Fig. 1).

Key concepts in the reconstruction of the parotid region are minimizing skin tension to obtain fast and better healing of the surgical site, minimizing skin mismatch in terms of skin color and texture, restoring volume and facial contour in the case of deep resections and bone resections, and restoring facial nerve function if compromised. With these goals in mind, a structured reconstructive decision-making framework was developed, as shown in Fig. 1.

The analysis of these parameters is used to determine, in each case, whether a complex reconstruction is necessary and, if so, which reconstruction technique is the most appropriate. The choice of the best reconstructive option depends on two main factors: the characteristics of the deficit and individual patient variables. Advanced age, comorbidities, history of surgery, smoking habits, and previous head and neck radiotherapy can represent an obstacle to microsurgery, by making the patient high risk for a long procedure, or by presenting the microsurgeon with the difficulty of a vessel-depleted neck.

### Skin and soft tissues defects

Within the analyzed sample, 11 patients needed a skin and/or soft tissue resection to guarantee oncologic margins. The main variables that needed to be analyzed in skin and soft tissue defects were already outlined in the algorithm presented in Fig. 2.

Skin defects, for which superficial coverage is the reconstructive need, are further divided into small, medium and wide according to Dobratz and Hilger’s classification of skin cheek defects [13, 14, 16].

As for the small defects (< 1 cm), the best option is primary closure; for medium defects (2–3 cm), we recommend direct closure if possible without tension, or if not via local advancement or rotation flaps [14, 16].

Wide defects (> 3 cm) can preferably be repaired with local rotation or advancement flaps, and if the defect is too great, with a locoregional or a free flap.

Primary closure or use of local flaps ensures an optimal aesthetic result provided by a perfect cutaneous match in both color, thickness, and texture. Primary closure and local flaps also allow for surgical scars to be camouflaged by being placed in wrinkles and natural skin lines, parallel to relaxed skin tension lines [16–18]. Furthermore, the donor site morbidity is minimal, and the risk of ischemia is reduced since the skin maintains its original vascularization, therefore healing of the surgical wound is rapid and allows for a patient to more quickly begin postoperative radiotherapy if needed. The possibility of performing a primary closure or repairing the defect with local flaps depends on the dimensions of the defect, its position on the face, and the laxity of the surrounding skin.

Free flaps are generally reserved for cases of very large or composite skin deficits. The aesthetic result will be less satisfactory as the skin will not have a similar color match or texture, and scars will be more visible.

In the case of extensive soft tissue resection, resulting in a significant volume deficit, volume-providing flaps are used to restore facial contour and symmetry. These include the pectoralis major muscle flap and microsurgical free flaps, such as the ALT flap and the latissimus dorsi muscle flap, which will restore facial contour but have the downside of skin color and texture mismatch. The choice between free and pedicled flap depends on the patient’s characteristics, comorbidities, and personal choices. Aesthetic revisions can be performed after all treatments to better hide scars, and recontour a bulky flap if desired by the patient.

In our series, soft tissue reconstruction provided satisfactory restoration of facial contour in most patients, with acceptable facial symmetry at rest during clinical follow-up. Minor wound healing issues were occasionally observed but were managed conservatively, and reconstruction did not significantly delay the initiation of adjuvant therapy.

### Bone defects

While bone resection does not always determine an impairment in facial aesthetic and function, primary closure or locoregional dermo-epidermal flaps are rarely sufficient. If the bone resection is large enough that it causes significant alterations in form or function, reconstruction is mandatory. These alterations could involve the dental occlusion, mastication, phonation, mandibular movements, and facial symmetry.

As described in the algorithm shown in Fig. 3, our preferred reconstructive option is represented by free bone flaps, particularly the iliac crest flap, which was used in two patients. Among the advantages of this flap are its versatility, the reduced morbidity of the donor site and the excellent aesthetic and functional results, and due to the significant bone stock it provides, it is ideal for placement of dental implants either during the primary surgery, or as a secondary procedure [19]. In the present cohort, restoration of mandibular continuity was achieved in all patients undergoing bony reconstruction. During follow-up, patients recovered adequate oral competence and mastication, and when oncologically appropriate, were referred for prosthetic rehabilitation.

In the case of a contraindication to the use of a free flap, or in cases of patient refusal, a satisfactory option is the pedicled locoregional pectoralis major muscle flap, which was used in three patients. As described above, the aesthetic result will be less satisfactory, but it guarantees the recovery of function (chewing, swallowing, phonation, separation of the oral cavity from the neck). It has a low rate of postoperative complications and a relatively short recovery time, making it suitable for patients indicated for post-operative radiotherapy [20].

Other options, especially when an extensive skin and soft tissues loss is associated with bone resection, are the ALT flap and the latissimus dorsi flap, due to the significant tissue bulk one can harvest with either flap.

Results of the present study agree with the published literature, in that it demonstrated that the choice between free and pedicled flap is mainly guided by the age and general conditions of the patient [21], and by their need to undergo post-operative radiotherapy. If radiotherapy is indicated, it is imperative to opt for the surgical choice that guarantees faster cure rates and lower complication rates, to allow a faster oncological treatment. An analysis of the data collected in the study shows a substantial difference in the mean age of patients undergoing reconstruction with microsurgical free flaps (43 ± 7) and patients undergoing reconstruction with pedicled flaps (63 ± 7).

### Facial nerve defects

Since the main aim of surgery is achieving an oncologically sound resection, there are two basic indications for facial nerve resection: neoplastic infiltration of the nerve or when it is unable to be separated from the neoplasm [1, 6].

Preoperative paralysis of the nerve is a strong indication for neoplastic infiltration [22]. The incidence of facial palsy by parotid cancer at the time of presentation is 12–15% [11, 23].

In all other cases it is imperative to pay the utmost attention to nerve preservation during parotid resection, whatever the resection technique chosen may be.

Our experience results in a preference for immediate nerve reconstruction over delayed reconstruction, to avoid scarring of the nerve stumps and denervation and atrophy of the muscles. Both neurorrhaphy and nerve grafts were used, and both have adequate resistance to post-operative radiotherapy [1]. In our experience, patients undergoing immediate facial nerve reanimation showed partial recovery of voluntary facial movement during clinical follow-up, with improvement in facial symmetry at rest and during expression. Static reanimation procedures contributed to better eyelid closure and oral competence, although active facial movement remained limited in these cases.

As illustrated in Fig. 4, reconstruction is possible if distal branches of sufficient diameter and length are available. If the main nerve trunk is available, the best option is restoring the continuity of the nerve trunk, through neurorrhaphy of the nerve stumps or with interposition of a nerve graft (if the gap length is more than 2 cm) [1, 23–25].

Restoration of facial nerve continuity has the advantage of preserving spontaneous and emotional facial movements.

When the proximal stump of the nerve is not available, but the distal branches are found, an alternative source of reinnervation is chosen: the masseteric nerve and the hypoglossal nerve are the most frequent choices.

The main disadvantage of this technique is the loss of spontaneous and emotional movement. However, the use of both the masseteric nerve and the hypoglossal nerve as donors guarantees a rapid and effective functional recovery, by virtue of the high number of myelinated fibers, easy rehabilitation of the patient, and low morbidity at the donor site.

After an initial period of nerve recovery, the patient will be directed to physiotherapy, where he/she will be taught how to obtain voluntary movement (e.g., to smile, to clench the teeth in case of use of the masseteric nerve, or to push the tongue against the hard palate in case of use of the hypoglossal nerve). With time, and with continuous physiotherapy, it is possible in some cases to regain spontaneous, emotional movement, due to cortical adaptation that will eventually make the act of smiling spontaneous.

If the nerve resection occurs at the level of the smaller, distal branches of the nerve, reconstruction will not be possible. In this case, static procedures will be used to improve function (lid closure, oral competence) and aesthetics (partial recovery of facial symmetry). For delayed reconstruction of the facial nerve, the use of reinnervated muscle flaps may be considered.

Static procedures are useful both in combination with nerve reconstruction to bridge the recovery period or can be carried out in isolation when a reconstruction of the nerve is not possible. They include upper eyelid lipofilling, upper eyelid tarsorrhaphy, and upper eyelid gold weight implantation to support eye closure. Static suspension of the naso-labial sulcus to the zygomatic arch periosteum using fascia lata grafts or nylon sutures can assist in camouflaging facial asymmetry [26].

A contraindicated technique in the case of reconstruction of the VII cranial nerve for malignant neoplasia of the parotid gland, is the technique of reconstruction of the nerve by neurorrhaphy, with interposition of a cross-face graft between the residual healthy peripheral branches and a redundant branch of the contralateral facial nerve. This is because some of the malignant salivary gland tumors have the characteristic of being strongly neurotropic and in case of recurrence of the neoplasm, it could easily invade the graft and expand into the contralateral parotid gland.

After the parotidectomy and reconstruction surgery, ancillary measures such as lipofilling can be taken to improve the aesthetic result.

The limitations of this study include the relatively small sample size and its retrospective design, which did not allow uniform collection of standardized functional outcome measures, such as detailed House–Brackmann grading, in all patients. Functional recovery was assessed clinically during follow-up visits, focusing on voluntary facial movement, symmetry at rest, eyelid closure, and oral competence.

Our reconstructive approach aligns with and expands upon prior literature in the field of parotid oncologic surgery. In their study of 58 patients, Cai et al. [8] proposed a repair strategy based on anatomic subunits and depth of invasion, underscoring the value of defect-driven planning. Dobratz and Hilger [16] similarly stressed the importance of topographic analysis in cheek and parotid reconstructions, integrating aesthetic subunits with functional restoration.

Large-scale analyses have confirmed the heterogeneity of reconstructive strategies following parotidectomy. Bovenzi et al. [27], analyzing over 11,000 cases, highlighted the influence of tumor characteristics and patient comorbidities on reconstruction choice, as well as the frequency of complications such as wound healing delay and salivary fistula. Dou et al. [28] proposed a structured algorithm stratified by defect dimension and tissue type, which closely mirrors the rationale of our proposed framework and supports reproducibility in routine clinical settings.

Further studies have addressed specific technical challenges. Mangialardi et al. [29] demonstrated the feasibility of chimeric scapulodorsal free flaps to simultaneously restore soft tissue volume and facial nerve function in extensive oncologic defects. Wang et al. [30] validated the use of superficial circumflex iliac perforator (SCIP) flaps for moderate-volume reconstructions, combining low donor-site morbidity with reliable vascularity. Xiao et al. [31] emphasized the need for robust reconstruction in the context of biologically aggressive parotid carcinomas, such as primary squamous cell carcinoma, in order to avoid delays in adjuvant treatment and preserve function. Finally, Ch’ng et al. [32] offered a detailed classification of post-parotidectomy defects and corresponding reconstructive strategies, highlighting the role of both pedicled and free flaps in complex reconstructions.

Collectively, these studies support the need for flexible, algorithm-based reconstruction tailored to defect characteristics, patient status, and oncologic priorities—an approach consistent with our clinical experience over two decades.

## Conclusions

Reconstructive surgery has dramatically expanded surgical indications for parotid malignancies, by making it possible to resect large, locally advanced, infiltrating tumors. Reconstructive surgery of the head and neck has as its main goals restoring function and aesthetics, as well as to ensure a fast recovery to allow select patients to undergo adjuvant treatment (mostly radiation therapy) as soon as possible after surgery.

Restoration of facial contour and symmetry and the abilities of speech and mastication represent a key point in the treatment of head and neck malignancies, as it means a better quality of life for the patient.

To date, there are no standardized guidelines in international literature for parotid region defects, nor a clear shared algorithm to which the surgeon can refer when deciding on the type of reconstruction to perform. The purpose of this study is to display the results of ten years of experience of our institution in the treatment of parotid gland malignancies and in the reconstruction of the defects resulting from the ablative surgery, in the attempt to suggest recommendations for the best reconstructive option for every case. The suggested techniques vary from first and most simply, primary closure and the use of pedicled, to more advanced techniques like microvascular free flaps. To this end, we show how in our daily practice we apply a structured decision-making approach to the deficit expected to result from ablative surgery. Because of this a reconstruction tailored to the defect can be designed, bearing in mind the objectives of the reconstructive surgery, namely restoration of function and facial contour balanced with rapid healing of the surgical site, if the patient needs to undergo radiation therapy.

## Data Availability

The datasets generated and analyzed during the current study are available from the corresponding author on reasonable request.
